# *In Silico* Screening of Putative *Corynebacterium pseudotuberculosis* Antigens and Serological Diagnosis for Caseous Lymphadenitis in Sheep by Enzyme-Linked Immunosorbent Assay

**DOI:** 10.1155/2021/9931731

**Published:** 2021-07-31

**Authors:** Daniela Droppa-Almeida, Caroline de Santana Ferreira, Ioná Brito, Sibele Borsuk, Jorge Alberto López Rodríguez, Isabel Bezerra Lima-Verde, Francine Ferreira Padilha

**Affiliations:** ^1^Laboratório de Biomateriais, Instituto de Tecnologia e Pesquisa, Universidade Tiradentes, Aracaju, Sergipe, Brazil; ^2^Laboratório de Biotecnologia Infecto-parasitária, Centro de Desenvolvimento Tecnológico, Universidade Federal de Pelotas, Pelotas, Rio Grande do Sul, Brazil; ^3^Laboratório de Biologia Molecular, Instituto de Tecnologia e Pesquisa, Universidade Tiradentes, Aracaju, Sergipe, Brazil

## Abstract

*Corynebacterium pseudotuberculosis* is the etiologic agent of Caseous Lymphadenitis (CLA), a disease leading to severe damage in sheep and goats farming due to the lack of serological diagnosis, treatment, and effective prophylaxis. In this context, several strategies in an attempt to discover new antigens to compose diagnosis assays or vaccines are fundamental. Therefore, this study aimed to use bioinformatics software to evaluate the critical chemical characteristics of unknown proteins of *C. pseudotuberculosis* by selecting them for heterologous expression in *Escherichia coli*. For this purpose, six protein sequences of ascorbate transporter subunit, UPF protein, MMPL family transporter, Ribonuclease, Iron ABC transporter domain-containing permease, and fimbrial subunit were obtained. *In silico* analyses were performed using amino acid sequences to access immunodominant epitopes and their antigenic and allergenic potential and physicochemical characterization. The expressed proteins were used as an antigen for serological diagnosis by ELISA. All proteins showed distinct immunodominant epitopes and potential antigenic characteristics. The only proteins expressed were PTS and Ribonuclease. In parallel, we expressed CP40 and all were used with ELISA antigen in 49 CLA positive sera and 26 CLA negative sera. The proteins alone showed 100% sensitivity and 96.2%, 92.3%, and 88.5% specificity for rPTS, rRibonuclease, and rCP40, respectively. When proteins were combined, they showed 100% sensitivity and 84.6%, 92.3%, 88.5%, and 92.3% specificity for rPTS/rCp40, rRibonuclease/rCP40, rPTS/rRibonuclease, and rPTS/rRibonuclease/rCP40, respectively. The results of this study show an excellent correlation of sensitivity and specificity with all proteins. None of the specificity values preclude the potential of rPTS, rRibonuclease, or rCP40 for use in ELISA diagnostic assays since the results of this work are superior to those of other studies on CLA diagnosis described in the literature.

## 1. Introduction

Caseous Lymphadenitis (CLA) is a chronic infectious disease that affects small ruminants. This pathology is a consequence of infection by a Gram-positive coccobacillus known as *Corynebacterium pseudotuberculosis*. Moreover, the formation of pyogranulomas in lymph nodes and internal organs represents its chief characteristic [1, 2]. As a consequence, CLA presents two clinical presentation forms: the external one, which is responsible for the majority of the cases and mainly affects skin and external lymph nodes, and the internal one, denominated asymptomatic since it affects lymph nodes and internal organs such as lungs, kidneys, liver, and spleen [3].

Due to the vast spectrum of hosts, several cases of CLA are present in many countries, for example, Australia, New Zealand, South Africa, the United States, Canada, and Brazil, and most of the cases are related to small ruminants [4, 5]. The primary strategy for CLA control is based not only on vaccination but also on the adoption of complementary measures related to the treatment or eradication of sick animals and specialized care in routine handling [6]. Although these vaccines generate an immune response, which leads to the production of specific antibodies, their efficiency is still discussed [7]. Another factor that contributes to the dissemination of the disease is the lack of diagnosis for cases of internal CLA.

The diagnosis of asymptomatic animals is an important factor for the effective control of CLA, as many animals develop the visceral form of the disease or are still in the initial stage of infection [8]. The current diagnosis to detect *C. pseudotuberculosis* is based mainly on the clinical examination of the lesions and their identification by phenotypic and biochemical tests due to the variability of the characteristics of the genus *Corynebacterium* [9, 10]. Recently, it has been suggested that the use of the Polymerase Chain Reaction (PCR) technique based on the 16S rRNA genes [11], phospholipase *D* (PLD), and rpoB, used together [12], resulted in tests of high sensitivity, reproducibility, and diagnostic efficiency; however, they also need the initial clinical examination.

Because of the limited diagnostic methods available, there is a need to use serological tests, in particular, the ELISA technique (enzyme-linked immunosorbent assay) that is effective in detecting all cases of CLA, asymptomatic or not. Some ELISAs have already been developed for this purpose, using different antigens such as cell preparations and proteins. However, the levels of sensitivity and specificity are still unsatisfactory [13, 14].

Thus, studies are conducted regarding the importance of exoproteins in the pathogen-host interaction to identify new molecular targets to develop effective diagnostic tests with significant levels of sensitivity and specificity [15]. In this way, the sequencing of several genomes of *C. pseudotuberculosis* [16, 17] contributed to finding potentially viable antigens before performing experiments to verify them. This technique increases the speed of antigen identification, decreases costs, increases vaccine strategies to obtain potential antigens for serological diagnosis. Many bioinformatics tools are available to facilitate this process, and the success of this method will depend on the accuracy and prediction of antigens. According to the literature, other studies have been carried out to obtain antigens.

A study performed by Droppa-Almeida et al. [18], in which they used CP40 sequences to obtain immunodominant epitopes, which presented the potential to formulate a multiepitope vaccine, showed a significant interaction of peptides with essential receptors of innate immunity. Similarly, Santana-Jorge et al. [19] verified *in silico* the following virulence factors Spac, PknG, and NanH, which presented a considerable potential for the development of vaccines against CLA.

In addition to the design of vaccines, the *in silico* search for potentially antigenic targets and subcellular location among other analyses become important in the search for antigens to compose serological diagnoses. Faria et al. [20] used the bioinformatics tools to predict immunodominant epitopes, approximately 50 proteins, from *Leishmania infantum* that obtained synthetic peptides that served as antigens for serological diagnosis. With this, it is possible to realize that the bioinformatics tools can act in several aspects, either in the identification of proteins of interest or in the design of synthetic peptides, both possible to compose vaccine formulation or an antigen for serological diagnosis.

Thus, because of the number of putative proteins of *C. pseudotuberculosis*, ten of these proteins were selected using bioinformatics tools, and two were expressed in *E. coli* to compose antigens for the serological diagnosis of CLA.

## 2. Methodology

**Figure 1:**
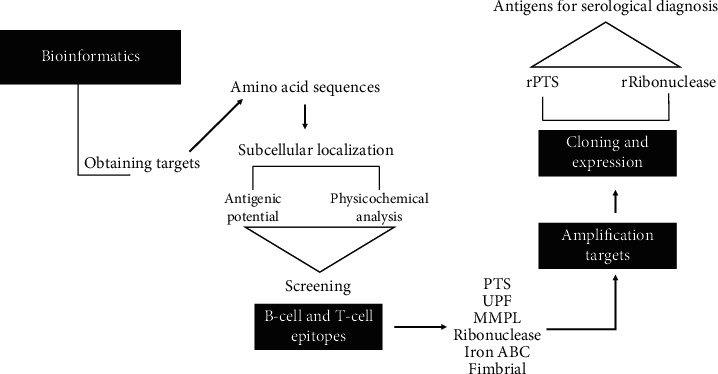
Illustrative image of the methodology used in sequence. Firstly, bioinformatics analyses were performed for the selection of important proteins of *Corynebacterium pseudotuberculosis*. After analyses, regarding the signal peptide, presence of antigenicity, physicochemical characterizations, and presence of immunodominant epitopes, a total of six proteins were selected and followed for PCR, cloning, and expression. Only two of the selected proteins were expressed in *E. coli*.

The methodology follows the illustrative scheme as shown in Figure 1.

### 2.1. Amino Acid Sequences

The amino acid sequences of the target proteins were retrieved from NCBI GenPept: ascorbate transporter subunit (PTS) (WP_058831991), UPF protein (AEP69811), MMPL family transporter (WP_013240996), Ribonuclease (WP_014300499.1), Iron ABC transporter domain-containing permease (ATV80741), and fimbrial subunit (ADK29694.1).

### 2.2. Subcellular Localization, Prediction of Protective Antigens, and Physicochemical Properties

#### 2.2.1. Prediction of Signal Peptides and Protein Localization

The software SignalP 4.1 available at http://www.expasy.org/proteomics was used for the verification of the signal peptides and, consequently, its location in *C. pseudotuberculosis*. This tool contains two types of neural networks, where it is possible to select sequence options with or without transmembrane sets. All analyses performed with Gram-positive bacteria used the two neural networks [21]. Then, the amino acid sequences were evaluated by PSORTb 3.0.2 [22] to predict the subcellular localization of the target proteins.

### 2.3. Antigenic Potential

To verify the antigenicity of proteins and peptides previously predicted, VaxiJen30 v2.0 (http://www.ddg-pharmfac.net/vaxijen/VaxiJen/VaxiJen.html) was employed. This software works based on independent alignment prediction of protective antigens. Additionally, it was developed to allow the classification of antigens according to the physicochemical properties of proteins without resorting to sequence alignment. Also, the server accuracy ranges from 70% to 89%, dependent on the target organisms [23].

### 2.4. Physicochemical Analysis

Different physicochemical properties for protein vaccines were studied, including molecular weight (MW), isoelectric point (pI), instability index, in vitro and in vivo half-life and aliphatic index, and the large mean of hydropathicity (GRAVY). All these properties were evaluated using the ProtParam server at http://web.expasy.org/protparam [24].

### 2.5. B-Cell Epitope Prediction

Linear B-cell epitopes were predicted from the target protein sequences using the following software: BCPreds, which is also based on machine learning methods but involves those that apply to support vector machines (http://ailab.cs. iastate.edu/bcpreds/). Currently, BCPreds works selecting three methods of prediction: (i) AAP implementation method [25]; (ii) BCPred (EL-Manzalawy et al., 2008); (iii) FBCPred [26]. The chosen method was FBCPred, which works by choosing peptides with nine amino acid residues.

### 2.6. Amplification of Targets

The PCR molecular technique amplified the genes corresponding to each protein through specific primers designed by Vector NTI 11 software (Invitrogen) (Table 1). The PCR amplification reaction was performed to a final volume of 50 *μ*L containing genomic DNA of *C. pseudotuberculosis* strain 1002 (50 ng), primers (10 *μ*M), autoclaved MiliQ water (22 *μ*L), and Mastermix (Promega) (25 *μ*L) containing Taq DNA polymerase, dNTPs, and MgCl_2_ under the following conditions: initial denaturation at 94°C (5 min), followed by 30 cycles corresponding to denaturation at 94°C (1 min), annealing at 55°C (1 min), extended at 72°C (1.5 min), and with a final extension of 72°C (7 min) in a thermocycler (Mastercycler Gradient, Eppendorf, Germany). The amplicons were analyzed on a 1% agarose gel stained with Blue Green (LGC Biotechnology).

### 2.7. Cloning Procedures and Recombinant Proteins Expressed in *Escherichia coli*

The gene-related amplicons were cloned into the pAE expression vector [27], according to the restriction sites established for each ORF (Open Reading Frame), as indicated in Table 1. For this purpose, each amplification product and the vector were digested with the respective restriction enzymes. Then, both were ligated at room temperature in a solution containing 1 *μ*l of the T4 DNA ligase enzyme (Invitrogen), 0.5 *μ*l of the vector, 2 *μ*l of buffer, and 15.5 *μ*l of MiliQ H_2_O. The products of the constructs were transformed into *E. coli* TOP 10 electrocompetent cells and seeded on plates containing LB medium with ampicillin (100 *μ*g/ml) and incubated for 16 h at 37°C. The obtained colonies were submitted to screening by lysis with phenol-chloroform (v/v) and analyzed in 1% agarose gel. The selected clones were cultured in LB broth with ampicillin (37°C/140 rpm for 12–16 h) before extracting the plasmid DNA using the Plasmid Miniprep Spin kit (GE Healthcare Life Sciences). Confirmation of the presence of each gene in the recombinant clones was carried out by digestion with the restriction enzymes used in cloning the pAE vector.

### 2.8. Recombinant Protein Expression

Constructs with the genes of interest were transformed into *E. coli* BL21 Star strain by heat shock. Besides, the expression of the recombinant proteins was induced with 1 mM Isopropyl *β*-D-1-thiogalactopyranoside (IPTG) in each culture under orbital shaking (37°C/3 h). Finally, Western Blot confirmed the expression of the recombinant proteins (36).

### 2.9. Western Blot for Confirmation of Protein Expression

Samples from the *E. coli* culture, which had been transformed by recombinant plasmids, were added to the sodium dodecyl sulfate (SDS) buffer and beta-mercaptoethanol. Then, after heating at 100°C for 10 min, the samples were electrophoresed on 12% polyacrylamide gel. The proteins were then electroblotted onto a nitrocellulose membrane (GE Healthcare Life Sciences). The membrane was blocked with 5% skimmed milk in PBS (1h/37°C) and then washed with PBS 0.05% Tween (PBS-T) (3 times for 5 min each), before being incubated with the anti-6X-his monoclonal antibody 1 : 4000 (Sigma) (1h/37°C). After three membrane washes with PBS-T, peroxidase-conjugated anti-mouse IgG antibody (Sigma), diluted in PBS-T (1 : 4000), was added. Finally, the incubation process (1h/37°C) was performed before revealing it with 3,3′-diaminobenzidine (DAB) and H_2_O_2_.

### 2.10. Recombinant Protein Purification

After centrifugation, a *pellet* was obtained (14000g/15 min/4°C). For its solubilization, it was resuspended in 40 mL of wash solution (200 mM NaH_2_ PO_4_, 500 mM NaCl, 5 mM imidazole, and 8M urea pH 8.0) by adding 100 mg/mL lysozyme followed by the sonication method (5 cycles of 15s, 20 kHz). In this procedure, the solubilization solution was kept under stirring at 4°C for 16 h. The proteins were then filtered in a 0.45 *μ*m membrane before purification by nickel-loaded Sepharose column affinity chromatography (HisTrap™ GE Healthcare Life Science). The protein preparation was then dialyzed (cellulose bags, 25  nm × 16  nm, Sigma) in 1x saline phosphate buffer (PBS)/0.2% urea, with two daily exchanges (7 days/4°C) for desalting. Furthermore, protein quantification was performed using BCATM Protein Assay Kit (Pierce Chemical Company).

### 2.11. Characterization of Antigenicity of Recombinant Proteins by Dot Blot

To verify antigenicity 5 *µ*L of each protein (rRibonuclease, rPTS *e* rCP40) was deposited on the nitrocellulose membrane (GE Healthcare Life Sciences). After drying (15 min/room temperature), the membrane was a block with PBS plus 5% skim milk (1 h/room temperature) before being incubated with negative and positive sheep to CLA (1 : 50 in PBS-Tween 20% for 1 h/37°C). After this step, the membrane was incubated with IgG sheep secondary antibody (1 : 4000 em PBS-T) conjugated with peroxidase (Sigma) (1 h/37°C). The reaction was revealed with 3,3′- diaminobenzidine (DAB) and H_2_O_2._.

### 2.12. Sheep Serum

The sheep serum sample was collected in Nossa Senhora da Gloria city, Sergipe. 75 sheep serum sample total were used in this study, 49 positive to CLA, with abscess, and these cases were confirmed with *C. pseudotuberculosis* with API coryne Kit and 26 negatives to CLA from neonate sheep, uninfected and isolated from the herd. The negative serum was used with standard to evaluate the cut-off point of the ELISA test about the recombinant proteins tested [28].

### 2.13. ELISA with Secreted Antigen from Strain 1002 *C*. *pseudotuberculosis*

To this assay, secreted antigen from 1002 strain *C. pseudotuberculosis* was cultured in BHI broth for 48h with standard to confirmed the sample serum positivity and serum negativity according to the methodology described by Seyffert et al. (2010) [28] with modifications. The immunoassay by ELISA 96-well plates, high binding, were sensitized with secreted antigens in the proportion 1 : 100 each well in Carbonate-Bicarbonate Buffer (0.05M, pH 9,6) and incubated for 16 h at 4°C. Subsequently, the plates were washed 3 times with PBS-T (PBS 1X, pH 7.4, 0.1% tween 20) and blocked with 100 µL/well of 5% nonfat milk in PBS (1 h at 37°C). Then, the plates were washed again, and 100 µL/well of sheep serum (1 : 50 in PBS-T) was added in duplicate in the plate. After 1 h of incubation at 37°C and more washes, 100 *µ*L/well of anti-sheep antibodies conjugated to peroxidase (Whole IgG (Sigma)) was added (1 : 10.000). After five more washes, 50 *µ*L/well of TMB (Bio-Rad) was added, and the plates were incubated for 15 min at room temperature and in the dark to reveal the results. To stop the reaction, 25 *µ*L/well of H_2_SO_4_ solution (4N) was added. Optical density (OD) was determined with an automatic photocolorimeter for ELISA (Microplate Reader MR-96A, Mindray) at 495 nm.

### 2.14. ELISA with Proteins rPTS, rRibonuclease, and rCP40

Each recombinant protein was used individually and in the association. According to standardization, the individual assay used 100 ng of each protein and 1 : 50 of serum dilution, in association assay were used a total of 50 ng with protein mix and 1 : 100 of serum dilution.

### 2.15. Statistical Analysis

To evaluate the specificity, sensibility, predictive value, and cut-off point, the experimental data were analyzed by Receiver Operating Characteristic (ROC) with the software MedCalc statistical (version 10.3.0). This analysis aims to differentiate the true positives from the true negatives to establish the cut-off point. The evaluation of the sensitivity and specificity of the experimental data was done with each protein individually and in the association.

## 3. Results and Discussion

### 3.1. Subcellular Localization, Signal Peptide, and Antigenicity

Concerning the location of the proteins, their primary structure was first obtained before the online servers SignalP-4.1 and PSORTb were applied (Table 2). The former verified that none of the sequences present a peptide signal. However, the latter showed that most proteins are present in the cellular membrane, a site also interesting for use in vaccines or as an antigen for immunodiagnosis. Membrane proteins and secreted proteins are considered potential vaccine targets since they represent the host-pathogen interface. These proteins may interact more directly with host molecules for cell adhesion, invasion, multiplication, and evasion of the immune response [29, 30].

For the antigenicity profile, the Vaxijen server was used and the provided *z* descriptors indicate the most relevant physicochemical properties for antigen recognition. The value of *z* has a threshold of 0.4 [23]. Given this threshold and the obtained data, the antigenicity of all the proteins explored in this work is described in Table 2.

### 3.2. Physicochemical Analysis

ProtParam server was used to check some character molecular weight, theoretical index, amino acid composition, atomic composition, extinction coefficient, instability index, aliphatic index, and the large mean of hydropathicity (GRAVY) as described in Table 3. This parameter is the point at which the total net charge of the amino acid or protein molecule is zero. These analyzes are based on equilibrium constant points, which means that the protein is stable at pH corresponding to one described in Table 3. All proteins demonstrated a stable profile and the high aliphatic index indicates protein stability at various temperatures. Moreover, when the GRAVY value of hydropathicity is negative the proteins are hydrophilic; in other words, they have significant interaction with water molecules.

*C. pseudotuberculosis* demonstrates interesting survival mechanisms and can use different strategies to adapt to its environment. Once it is successfully established within a host, it can replicate within the phagocytic cells. This pathogen will then easily block the immune system. As a result, chronic infections can last for most, if not all, of an animal's life [31]. Chronicity leads to the formation of pyogranulomas intended to protect the animal from possible generalized infection. Currently, bacteria are known to bypass the pyogranuloma cell barrier to infect other parts of the host organism. Because it is a facultative intracellular bacterium, the immune response against this pathogen is Th1 type since it will be assisted by CD4 + helper cells, macrophages, and B lymphocytes. When this type of response is activated, the production of IgG antibodies is increasing, mainly of the IgG2a and IgG2b isotypes, which signal a Th1 response [32]. By understanding the pathogenesis of CLA, it is possible to understand the type of vaccines that may present possible protection to the animals. The use of proteins as a vaccine antigen is increasing because it is an antigen whose response is dependent on T cells, activating both humoral and cellular response, important for facultative intracellular pathogens.

The use of potentiators, known as adjuvants, can aid in directing the response and may direct to Th1, Th2, Th9, or Th17 and these potentiators are crucial to the success of a protein antigen. This work presents six potential targets for the development of vaccines proteins for CLA and with important characterizations on each of them. In addition to the use of vaccine formulations, these proteins may be useful for the development of diagnostic kits for CLA since the diagnosis is still based on the culture and isolation of the bacteria present in the pyogranulomas found in the animals.

### 3.3. B-Cell Epitope Prediction

After analyzing the B-cell epitopes by the BCpreds software, a total of 18 epitopes for PTS, 45 for UPF, 26 for MMPL, 14 for Ribonuclease, 9 for IronABC, and 57 for Fimbrial were predicted. Immunodominant epitopes are specific regions of protein antigens that have binding to immunological receptors [33, 34]. Regarding B-cell epitopes, we turn to the production of antibodies, which can identify and neutralize antigens, such as viruses, bacteria, parasites, fungi, cancer cells, and some toxins, by binding to specific parts on their surface [35]. The paratope, a specific part of an antibody, binds to a particular region in the antigen that is called an epitope or antigenic determinant [36, 37]. Different from T-cell epitopes, most functional B-cell epitopes are conformational, although there are linear models as well [38].

Epitope databases consist of a collection of epitopes of pathogens such as viruses, bacteria, protozoa, and fungi. These databases have become an increasingly used source in the search for antigens for application in vaccines, in the diagnosis, and as immunotherapies [39]. Given the lack of vaccines and serological diagnosis for CLA, new target analyzes are essential, as well as the use of different strategies such as the use of synthetic peptides based on immunodominant epitopes. For this reason, these proteins were evaluated *in silico* and subsequently used to identify the immunodominant epitopes. The selection of the epitopes was aimed to contain nine amino acid residues since the B-cell epitopes are capable of both binding antibodies and B-cell receptors (BCR) [40]. Consequently, this condition will lead to the transformation of B cells in antigen-specific antibody-producing plasmocytes and consequently antibodies, proving to be important targets for the development of antigens for serological diagnosis.

## 4. Obtaining Recombinant Proteins

### 4.1. Amplification of Genes

**Figure 2:**
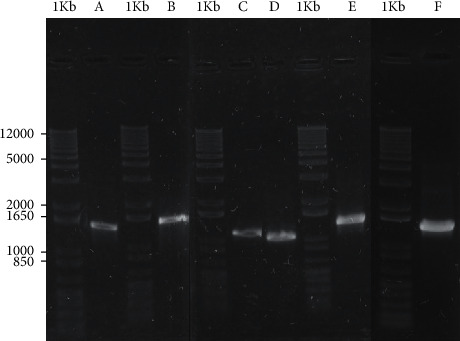
Agarose gel electrophoresis 1% of PCR amplification products of the ORFs of interest. 1 Kb : molecular size standard (1 Kb Plus DNA Ladder, Invitrogen); (A) amplicon of Upf; (B) amplicon of Mmpl; (C) amplicon of Ribonuclease; (D) amplicon of Iron; (E) amplicon of Fimbrial; and (F) amplicon of PTS. Cloning procedures and recombinant proteins expressed in *Escherichia coli*.

The amplification products of the genes, which were increased from the genomic DNA of *C. pseudotuberculosis* 1002 using primers described in Table 1, presented molecular sizes according to the *in silico* prediction.  Figure 2 shows the results of PCR amplification.

**Figure 3:**
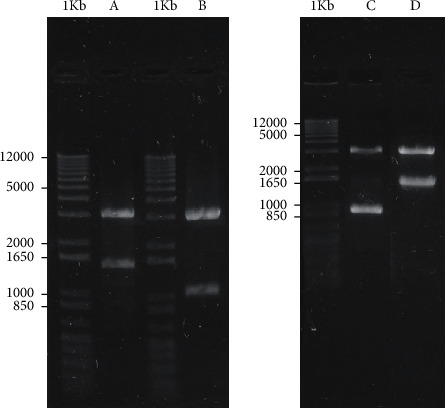
1% agarose gel electrophoresis of the characterization of the recombinant clones, after digestion with the respective restriction enzymes. 1 Kb : molecular size standard (1 Kb Plus DNA Ladder, Invitrogen); (A) pAE/PTS; (B) pAE/Ribonuclease; (C) pAE/CPIron; (D) pAE/CPFimbrial.

After the characterization of the recombinant clones, four clones were achieved: pAE/PTS, pAE/Ribonuclease, pAE/CPIron, and pAE/CPF-bound, as illustrated in Figure 3.

Regarding the *upf* and *mmpl* genes, although amplification products were obtained, after repeated attempts, no recombinant clones were achieved. One possible explanation for the failure to obtain plasmid constructs between these amplicons and the pAE vector may be an amplification error or in the synthesis of primer sequences.

**Figure 4:**
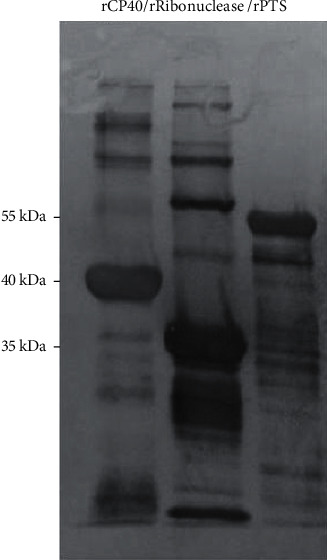
Confirmation of expression of the recombinant proteins rCP40, rRibonuclease, and rPTS, by Western Blot using anti-6Xhis.

Upon confirmation of the genes in the pAE vector, the pAE/PTS, pAE/Ribonuclease, pAE/Iron, and pAE/Fimbrial constructs were transformed by heat shock into the *E. coli* BL21 Star expression line. One colony of each recombinant clone was selected for this transformation and used for the expression of the respective proteins after induction with IPTG. Expression of the heterologous proteins was verified by Western Blot, presenting apparent molecular weights of approximately 55 kDa (rPTS) and 35 kDa (rRibonuclease), corresponding to the primary amino acid sequence of the proteins encoded by the selected genes, in addition to the six-histidines tail (Figure 4). rCP40 was used as a standard because of its frequent expression in the laboratory.

At this point, the fact that clones pAE/CPIron and pAE/Ribonuclease, when transformed in *E. coli* BL21 Star, did not express the respective recombinant proteins may be related to the presence of codons in the gene sequences of *C. pseudotuberculosis* that are little used by *E. coli*. The degree of frequency of some codons may be a limiting factor in the heterologous expression of proteins in expression lines [41].

Considerable amounts of rPTS and rRibonuclease are required for further studies on the role of these proteins in the pathogenicity and virulence of *C. pseudotuberculosis*. The use of these proteins for heterologous expression with *Escherichia coli* remains one of the most attractive uses among many systems available for protein production [18, 42]. Thus, *pts, upf, mmpl, Ribonuclease, iron*, and *fimbrial* genes were cloned into the same expression vector system and individually transformed into *E. coli* BL21 (DE3) strains. SDS-PAGE analyses show the successful expression of the rPTS and rRibonuclease proteins (Figure 4). As a result, these proteins can be used to compose both recombinant vaccines and serve as an antigen for immunoassay since there is a lack of serological diagnosis for CLA. From the present study, we suggest that various B-cell epitopes predicted from PTS, UPF, MMPL, Iron, Ribonuclease, and Fimbrial could be used for the development of a multipeptide vaccine to induce a complete immune response against *C. pseudotuberculosis*. The next step will be to experimentally evaluate these epitopes *in vitro* and *in vivo* to assess their true protective potential.

### 4.2. Antigenicity Characterization of the Recombinant Protein by Dot Blot

**Figure 5:**
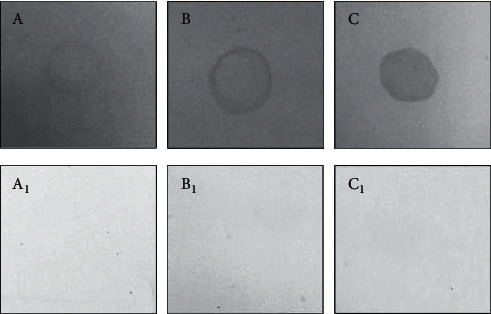
Dot blot to antigenicity characterization of recombinant protein. (A) rPTS evaluate with positive serum from CLA; (A_1_) rPTS evaluation with negative serum from CLA; (B) rRibonuclease evaluation with positive serum from CLA; (B_1_) rRibonuclease evaluation with negative serum from CLA; (C) rCP40 evaluation with positive serum from CLA; (C_1_) rCP40 evaluation with negative serum from CLA. Enzyme-Linked Immunosorbent Assay (ELISA) with secreted antigens of *C. pseudotuberculosis*.

To verify antigenicity from rCP40, rRiblonuclease, and rPTS proteins, sheep serums of positive and negative animals from CLA by Dot Blot were used.  Figure 5 shows the protein reactivity with positive serum, and it did not show with negative serum. This suggests the recombinant protein preserves the antigenic potential, even after heterology expression in *E. coli*. The Dot Blot is a technique widely used to determine the antigenic property of recombinant protein [43].

A total of 49 positive sera and 26 negative sera were used in indirect ELISA with secreted antigens of *C. pseudotuberculosis* 1002, and this assay was done to confirm the positivity and negativity of sheep sera. This methodology was validated and applied by Seyffert et al. [28] when carrying out a seroepidemiological study of the prevalence of CLA in the State of Minas Gerais, with a sensitivity of 93% and specificity of 98.5% in the detection of antibodies against antigens secreted by the referred bacteria.

**Figure 6:**
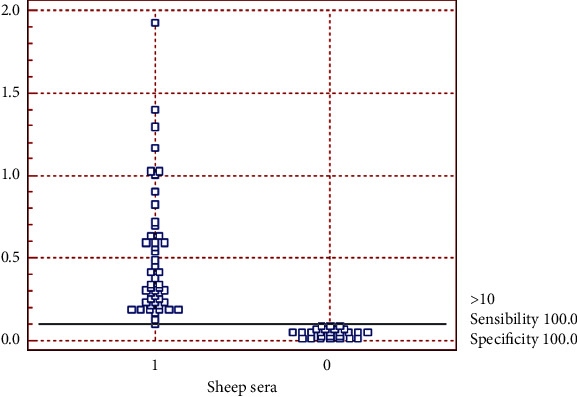
Indirect ELISA evaluation with antigens secreted from *C. pseudotuberculosis* 1002 with 75 sheep sera through the Receiver Operating Characteristic (ROC). (1) Positive serum samples; (0) samples of negative sera. This shows ELISA with rPTS, rRibonuclease, and rCP40 proteins.

Figure 6 shows the frequency distribution from these positive sera (1) and negative sera (0). The samples were positive when the cut-off showed a value of optical density (OD) _450n_ ≥ 0,10, referring to the mean ELISA absorbance values. There are no sera below the cut-off line at 1 or above the cut-off line at 0, so they indicate the absence of false negatives and positives, respectively.

**Figure 7:**
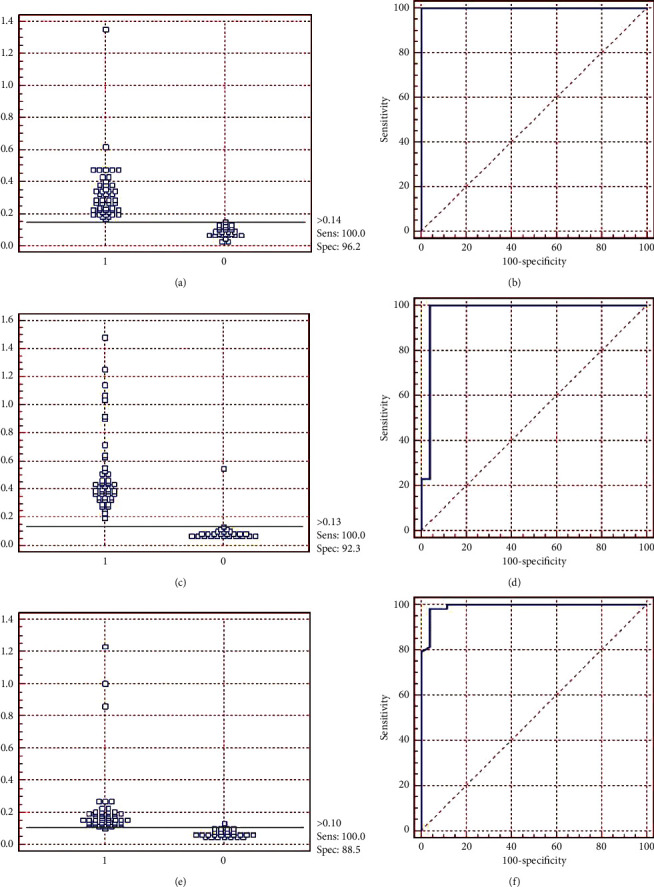
Indirect ELISA results with recombinant proteins (rPTS, rRibonuclease, and rCP40) as antigens, individually tested with 49 positive sera and 26 negative sera for CLA and ROC analysis of all assays. (a, b) ELISA rPTS with area under the curve (AUC): 1,000 and 95% confidence interval (CI): 0.951–1,000; (c, d) ELISA rRibonuclease with AUC: 0.970 and 95% CI: 0.902–0.955; (e, f) ELISA rCP40 with AUC: 0.991 and 95% CI: 0.934–0.997.

Figure 7 indicates the results of the ELISA using the rPTS, rRibonuclease, and rCP40 proteins individually. The samples were considered positive when the average absorbance value was higher than the cut-off point calculated for each of the proteins. The ROC analysis was used to determine the cut-off point, sensitivity, specificity, and predictive value for each of the tests. The values of these cut-off points were> 0.14 for rPTS, > 0.13 for rRibonuclease, and> 0.10 for rCP40. The sensitivity obtained was 100% for the three ELISAs (rPTS, rRibonuclease, and rCP40) and the specificity obtained was 96.2%, 92.3%, and 88.5% for rPTS, rRibonuclease, and rCP40, respectively (Table 4).

**Figure 8:**
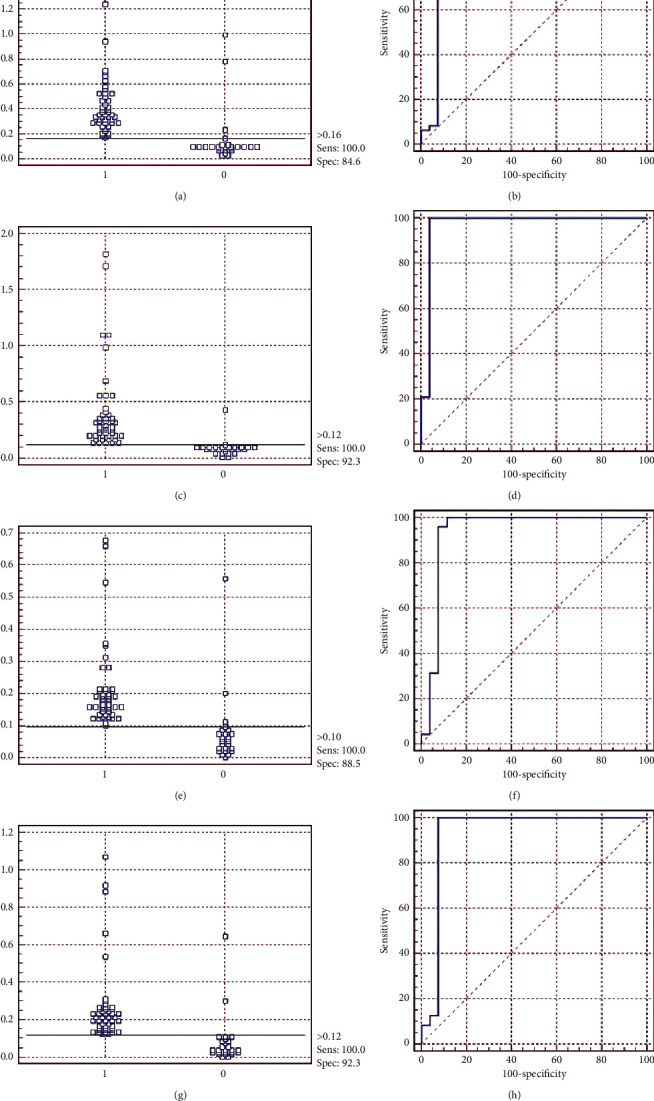
Result of indirect ELISA with recombinant proteins (rPTS, rRibonuclease, and rCP40) associated with each other, using 49 positive sera and 26 negative sera for CLA and ROC analysis of all ELISAs. (a, b) rPTS/rCP40 with AUC: 0.924 and 95% CI: 0.838–0.972; (c, d) rRibonuclease/rCP40 with AUC: 0.970 and 95% CI: 0.900–0.995; (e, f) rPTS/rRibonuclease with AUC: 0.935 and 95% CI: 0.853–0.979; (g, h) rPTS/rRibonuclease/rCP40 with AUC: 0.931 and 95% CI: 0.847–0.977.

On the other hand, when analyzing the results of the indirect ELISA tests where associations between proteins were used, sensitivities of 100% were observed, but varying specificities between those associations, as can be seen in Table 5 and Figure 8, together with their cut-offs.

The rPTS, rRibonuclease, and, rCP40 proteins were individually tested and in the association were impregnated in indirect ELISA to detect specifics antibodies of *C. pseudotuberculosis* in sheep sera, to develop an effective diagnosis for CLA in sheep. According to Bastos et al. [44], the search for tests that provide greater sensitivity in the diagnosis of CLA in sheep is due to the low sensitivity in tests already carried out on these animals. However, the results of this work indicated that ELISAs with recombinant proteins obtained 100% sensitivity in all tests, eliminating the probability of obtaining false-negative results in sheep.

However, about specificities, the values ranged from 84.6% to 96.2% (Tables 4 and 5). When comparing the values of ELISAs with individual proteins, it is observed that the specificities of the tests with associations are lower than that of rPTS (96.2%), even in the combinations in which this protein is present. It is noted that ELISAs with the associations rPTS/rRibonuclease and rPTS/rCP40 showed specificities of 88.5 and 84.6%, respectively, similar to that observed with rCP40 in the individual test.

Although they have variation in terms of specificity, the experimental results are promising, mainly because of the low sensitivities obtained, for example, by Rebouças et al. [45] when developing a diagnostic ELISA to detect gamma interferon (IFN-*γ*) as a marker of host cell-mediated immunity against the pathogen in sheep, resulting in a 100% specificity, however a low sensitivity of 55.8%. Binns et al. [46] used a cell preparation for the development of an ELISA that detected antibodies against *C. pseudotuberculosis* in the sera of sheep, obtaining a specificity of 100% but a sensitivity of 71%. Regarding the use of *C. pseudotuberculosis* culture supernatant to detect specific antibodies in sheep sera, Rebouças et al. [14] obtained a specificity of 99% and a sensitivity of 89%.

Concerning recombinant proteins, Menzies et al. [47] tested, over a year, an ELISA with recombinant PLD in experimentally infected goats, with a sensitivity of 81% and specificity of 97%. Still, regarding the rPLD protein, Sting et al. [48] correlated two ELISA tests (ELISA-whole cell antigens and ELISA-rPLD) to evaluate the seroprevalence of *C. pseudotuberculosis* in infected goats, where the first conferred a sensitivity of 81% and specificity of 98%, while the second sensitivity of 97% and specificity of 99%, thus showing that the result obtained by the ELISA with the recombinant protein was superior. However, according to a study by Hoelzle et al. (2013) [15], serological tests based on PLD as the only antigen are not able to identify all infected sheep since PLD was recognized in this experiment by only 70% of the positive sheep for CLA and by 100% of the positive goats.

Therefore, the results of this study show an excellent correlation of sensitivity and specificity (100% and 96.2%) for the rPTS test compared to other tests performed. However, all results are generally promising, as already mentioned above. None of the specificity values preclude the potential of rPTS, rRibonuclease, or rCP40 for use in ELISA diagnostic assays since the results of this work are superior to those of other studies on CLA diagnosis described in the literature.

Although Hoelzle et al. [15] have suggested that an ideal assay for the diagnosis of CLA is with the combination of two to three recombinant and immunodominant proteins of *C. pseudotuberculosis*, it should be noted that there are still no studies on the association of these proteins, this work being the pioneer in that sense. The rCP40 test with sheep sera showed a specificity of 85.5%, but in its association with the rPTS protein, the value was 84.6%, having reduced the individual potential of the rPTS (which is 96, 2%) and rCP40 itself. In the rRibonuclease/rCP40 test, which obtained a specificity of 92.3%, there was no variation in specificity compared to rRibonuclease. Finally, among the associations, the best results were those of the rRibonuclease/rCP40 and rPTS/rRibonuclease/rCP40 tests, which obtained 92.3% specificity, matching in numbers, to rRibonuclease.

Specificity results, although inferior and, in some cases similar to those of tests with individual proteins, do not compromise this approach. The associations proved to be positive. This fact is in line with tests performed for other diseases, such as Ferra et al. [49], who used the association between recombinant antigens successfully to diagnose *Toxoplasma gondii* infection in horses, pigs, and sheep, with sensitivity and specificity values of up to 100% depending on the species.

Still, at this point, Alizadeh et al. [50] developed an ELISA based on the association of two *Leptospira* recombinant proteins, which obtained 96.6% sensitivity and 83.2% specificity when compared to the gold standard used, is considered a useful technique for the diagnosis of leptospirosis. Faria et al. [20] also found a positive effect when using the association of *Leishmania* spp proteins—in diagnostic tests for leishmaniasis, obtaining sensitivity values of up to 75.81% and specificity of up to 95%, proving to be efficient.

According to the ROC analysis, the negative predictive value of all tests performed was 100%, regardless of the prevalence rate of herds. This reiterates the reliability of the ELISAs developed in the absence of false negatives because the greater the sensitivity of the test, the greater its negative predictive value, that is, the greater the probability of the animal being healthy in the face of the negative result.  Table 6 shows the predictive values of each indirect ELISA test, according to the average prevalence of 30% of CLA in the herd [51], as normally occurs in Brazil.

When analyzing the rPTS test, it showed the best positive predictive value about the other tests, showing that the proportion of infection among the positive animals detected through the test is high. This means that for every 100 positive tests, 91 sheep would be infected.

Therefore, the test's ability to identify true positives from true negatives is critical to any diagnostic test. In the specific case of CLA, this ability is a crucial factor in allowing the detection of apparently infected and asymptomatic animals, which are a source of the spread of the infection in the herd. Thus, an efficient and rapid diagnostic test allows adequate management of animals and the application of appropriate prophylactic measures, considering the socio-economic importance of sheep farming for the world.

## 5. Conclusion

The *in silico* analyzes demonstrated that *C. pseudotuberculosis* has several targets with the capacity for the development of vaccines and serological diagnosis. It was possible to determine six targets that presented *in silico* exciting results concerning their physicochemical characteristics and potential allergens and antigenic. Regarding immunodominant epitopes, there are numerous possibilities to compose diagnostic tests and multiepitope vaccines that could be able to differentiate vaccinated animals from naturally infected animals. Of the six proteins evaluated in this study, only two were expressed in *E. coli*, PTS, and Ribonuclease. With these recombinant proteins obtained, ELISA assays with positive and negative CLA sera were performed. CP40, which is an endoglycosidase that has already had its antigenic and immunogenic potential reported in the literature, was also used in this work. These three proteins were used alone and in combination, giving promising results in sensitivity and specificity. The difficulty in obtaining high specificities for serological diagnosis for CLA has already been reported in the literature due to the difference between hosts. In this work, it was evaluated only with sheep sera. With this, it is suggested new studies using these proteins in goat sera to verify the high specificity that these antigens presented in sheep, increasing the possibility of a serological diagnosis for CLA.

## Figures and Tables

**Table 1:** Primers for PCR and their respective restriction sites for binding to the vector pAE.

Base pairs	Primers	Description	Restriction sites
1542 (pb)	Forward: *pts*F	5′AGTCTGCAGAATGAACGTCCTACTTTC	*Pst*I
	Reverse: *pts*R	5′ CTAAACGTTTCACCCCAGTGGC	*Hind*III

1589 (pb)	Forward: *upf*F	5′- TAAGGATCCGCACGTGGC	*BamH*I
	Reverse: *upf*R	5′-CTAGAATTCCTATCCGCCTATAGTGT	*EcoR*I

1618 (pb)	Forward: *mmpl*F	5′- CTACTGCAGTCAGGGCGTAAT	*Pst*I
	Reverse: *mmpl*R	5′- AGGGAATTCAAATACCTGGTAG	*EcoR*I

1086 (pb)	Forward: *ribonuclease*F	5′- CTAGGTACCTCATGCCCACAGTATC	*Kpn*I
	Reverse: *ribonuclease*R	5′ CCTAAGCTTTTATTTCTTTCTTAAAGC	*Hind*III

969 (pb)	Forward: *iron*F	5′- ATAGGATCCTTGGTTGCATGCG	*BamH*I
	Reverse: *iron*R	5′-CTAGAATTCCTACCTTGGCCTTCTTG	*EcoR*I

1521 (pb)	Forward: *fimbrial*F	5′- ATAGGATCCGGCATCAAGGTAG	*BamH*I
	Reverse: *fimbrial*R	5′- ATAGGTACCAACCCGCAAAGAC	*Kpn*I

The underlined sequences correspond to the restriction site.

**Table 2:** Results about subcellular localization and antigenicity of the target proteins.

Proteins	Signal peptide	PsortB	Vaxijen score 0.40
PTS	No/*D* = 0.130 D-cutoff = 0.450	CMP score: 0.618	0.4699 (Probable Antigen)
UPF0182	No/*D* = 0.189 D-cutoff = 0.450	CMP score: 0.775	0.4806 (Probable Antigen)
MMPL	No/*D* = 0.357 D-cutoff = 0.450	CMP score: 0.663	0.6489 (Probable Antigen)
Ribonuclease	No/*D* = 0.137 D-cutoff = 0.450	CMP score: 0.484	0.6143 (Probable Antigen)
Iron	No/*D* = 0.402 D-cutoff = 0.450	CMP score: 0.554	0.4981 (Probable Antigen).
Fimbrial	No/*D* = 0.130 D-cutoff = 0.450	CMP score: 0.416	0.7775 (Probable Antigen).

CMP: cytoplasmic membrane protein.

**Table 3:** Physicochemical properties of the target proteins estimated using ProtParam.

Physicochemical property	PTS	UPF	MMPL	Ribonuclease	Iron	Fimbrial
Number of amino acids	513	896	795	361	340	995
Molecular weight	52613.17	98897.67	84887.14	39313.31	34979.52	108358.72
Theoretical pI	8.40	5.69	6.49	9.31	10.13	4.74
Total number of negative residues	22	101	62	27	13	148
Total number of positive residues	24	91	59	35	26	101
Instability index (II)	28.17	29.18	30.25	26.58	22.14	33.35
Aliphatic index	121.52	89.99	114.34	105.15	130.15	76.31
Grand average of hydropathicity (GRAVY)	0.884	−0.224	0.495	0.451	0.815	−0.474

The estimated half-life						
*In vitro*	30 hours	1.1 hours	30 hours	30 hours	30 hours	30 hours
*Yeast*	>20 hours	3 min	>20 hours	>20 hours	>20 hours	>20 hours
*Escherichia coli*	>10 hours	>10 hours	>10 hours	>10 hours	>10 hours	>10 hours

Negative residues: Asp + Gln; positive residues: Arg + Lys.

**Table 4:** Indirect ELISA results with recombinant proteins (rPTS, rRibonuclease, and rCP40) as antigens individually tested with 49 positive sera and 26 negative sera for CLA.

ELISA	Sensibility (%)	Specificity (%)
rPTS	100	96, 2
rRibonuclease	100	92, 3
rCP40	100	88, 5

**Table 5:** Indirect ELISA results with recombinant proteins associated and their sensibility and specificity.

ELISA	Sensibility (%)	Specificity (%)
rPTS/rCP40	100	84, 6
rRibonuclease/rCP40	100	92, 3
rPTS/rRibonuclease	100	88, 5
rPTS/rRibonuclease/rCP40	100	92, 3

**Table 6:** Positive and negative predictive values according to the prevalence of CLA.

Indirect ELISA (^*∗*^)	Prevalence of CLA (%)	Positive predictive values (%)	Negative predictive values (%)
rPTS	30	91, 8%	100
rRibonuclease	30	84, 7%	100
rCP40	30	78, 8%	100
rPTS/rRibonuclease	30	78, 8%	100
rPTS/rCP40	30	73, 5%	100
rRibonuclease/rCP40	30	84, 7%	100
rPTS/rRibonuclease/rCP40	30	84, 7%	100

^*∗*^Tests performed with recombinant proteins individually and in combination.

## Data Availability

The data from *in silico* and experimental analysis used to support the findings of this study are included within the article.
